# Healthcare trajectory of critically ill patients with necrotizing soft tissue infections: a multicenter retrospective cohort study using the clinical data warehouse of Greater Paris University Hospitals

**DOI:** 10.1186/s13613-022-01087-5

**Published:** 2022-12-20

**Authors:** Camille Windsor, Camille Hua, Quentin De Roux, Anatole Harrois, Nadia Anguel, Philippe Montravers, Antoine Vieillard-Baron, Jean-Paul Mira, Tomas Urbina, Stéphane Gaudry, Matthieu Turpin, Charles Damoisel, Djillali Annane, Jean-Damien Ricard, Barbara Hersant, Armand Mekontso Dessap, Olivier Chosidow, Richard Layese, Nicolas de Prost, AP-HP NSTI study group, Romain Arrestier, Michael Atlan, Thomas Bauer, Romain Bosc, Guillaume Carteaux, Cyril Charron, Bernard Clair, Yves Cohen, Jacques Duranteau, Muriel Fartoukh, Samy Figueiredo, Nicholas Heming, Jérémie Joffre, Raphaël Lepeule, Eric Maury, Lionel Nakad, Keyvan Razazi, Alain Sautet, Sébastien Tanaka, Léa Satre-Buisson, Emmanuel Weiss, Paul-Louis Woerther

**Affiliations:** 1 Service de Médecine Intensive Réanimation, Hôpitaux Universitaires Henri Mondor–Albert Chenevier, Assistance Publique-Hôpitaux de Paris (AP-HP), Créteil, France; 2 Groupe de Recherche Clinique CARMAS, Université Paris Est-Créteil, Créteil, France; 3 Service de Dermatologie, Hôpitaux Universitaires Henri Mondor-Albert Chenevier, Assistance Publique–Hôpitaux de Paris (AP-HP), Créteil, France; 4 Université Paris-Est Créteil Val de Marne (UPEC), Créteil, France; 5 Département d’anesthésie-Réanimation, Hôpitaux Universitaires Henri Mondor, Assistance Publique-Hôpitaux de Paris (AP-HP), Créteil, France; 6 Département d’anesthésie-Réanimation, Université Paris Saclay, Hôpital Bicêtre, Assistance Publique-Hôpitaux de Paris (AP-HP), Le Kremlin-Bicêtre, France; 7 Service de Médecine Intensive Réanimation, Hôpital Bicêtre, Assistance Publique-Hôpitaux de Paris (AP-HP), Le Kremlin-Bicêtre, France; 8 Department of Anesthesiology and Critical Care Medicine, Université Paris Cité, AP-HP, Hôpital Bichat-Claude Bernard; DMU PARABOL, Paris, France; 9 PHERE, Physiopathology and Epidemiology of Respiratory Diseases, French Institute of Health and Medical Research (INSERM) U1152, Paris, France; 10 Service de Médecine Intensive Réanimation, Hôpital Ambroise Paré, Assistance Publique-Hôpitaux de Paris (AP-HP), Boulogne-Billancourt, France; 11 CESP, UMR 1018, Université Paris-Saclay, Gif-sur-Yvette, France; 12 Service de Médecine Intensive Réanimation, Hôpital Cochin, Assistance Publique-Hôpitaux de Paris (AP-HP), Paris, France; 13 Service de Médecine Intensive Réanimation, Hôpital Saint-Antoine, Assistance Publique-Hôpitaux de Paris (AP-HP), Paris, France; 14 Service de Médecine Intensive Réanimation, Hôpital Avicenne, Assistance Publique-Hôpitaux de Paris (AP-HP), Avicenne, Bobigny, France; 15 Service de Médecine Intensive Réanimation, Sorbonne Université, AP-HP. Hôpital Tenon, DMU APPROCHES, Paris, France; 16 Service de Médecine Intensive Réanimation, Hôpital Tenon, Assistance Publique-Hôpitaux de Paris (AP-HP), Paris, France; 17 Service de Réanimation Polyvalente, Hôpital Antoine Béclère, Assistance Publique-Hôpitaux de Paris (AP-HP), Clamart, France; 18 Service de Médecine Intensive Réanimation, Université Paris Cité, AP-HP, Hôpital Louis Mourier, DMU ESPRIT, Colombes, France; 19 Service de Chirurgie Plastique Esthétique et Reconstructrice, Hôpitaux Universitaires Henri Mondor–Albert Chenevier, Assistance Publique-Hôpitaux de Paris (AP-HP), Créteil, France; 20 Unité de Recherche Clinique, Hôpitaux Universitaires Henri Mondor–Albert Chenevier, Assistance Publique-Hôpitaux de Paris (AP-HP), Créteil, France; 21 Equipe CEpiA (Clinical Epidemiology and Ageing), Université Paris-Est Créteil, INSERM, IMRB, Créteil, France

**Keywords:** Necrotizing skin and soft tissue infections, Healthcare trajectory, NSTI, Intensive care unit, Hospital mortality

## Abstract

**Background:**

Necrotizing skin and soft tissue infections (NSTIs) are rare but serious and rapidly progressive infections characterized by necrosis of subcutaneous tissue, fascia and even muscle. The care pathway of patients with NSTIs is poorly understood. A better characterization of the care trajectory of these patients and a better identification of patients at risk of a complicated evolution, requiring prolonged hospitalization, multiple surgical re-interventions, or readmission to the intensive care unit (ICU), is an essential prerequisite to improve their care. The main objective of this study is to obtain large-scale data on the care pathway of these patients. We performed a retrospective multicenter observational cohort study in 13 Great Paris area hospitals, including patients hospitalized between January 1, 2015 and December 31, 2019 in the ICU for surgically confirmed NSTIs.

**Results:**

170 patients were included. The median duration of stay in ICU and hospital was 8 (3–17) and 37 (14–71) days, respectively. The median time from admission to first surgical debridement was 1 (0–2) day but 69.9% of patients were re-operated with a median of 1 (0–3) additional debridement. Inter-hospital transfer was necessary in 52.4% of patients. 80.2% of patients developed organ failures during the course of ICU stay with 51.8% of patients requiring invasive mechanical ventilation, 77.2% needing vasopressor support and 27.7% renal replacement therapy. In-ICU and in-hospital mortality rates were 21.8% and 28.8%, respectively. There was no significant difference between patients with abdomino-perineal NSTIs (*n* = 33) and others (*n* = 137) in terms of in-hospital or ICU mortality. Yet, immunocompromised patients (*n* = 43) showed significantly higher ICU and in-hospital mortality rates than non-immunocompromised patients (*n* = 127) (37.2% vs. 16.5%, *p* = 0.009, and 53.5% vs. 20.5%, *p* < 0.001). Factors associated with a complicated course were the presence of a polymicrobial infection (adjusted odds ratio [aOR = 3.18 (1.37–7.35); *p* = 0.007], of a bacteremia [aOR = 3.29 (1.14–9.52); *p* = 0.028] and a higher SAPS II score [aOR = 1.05 (1.02–1.07); *p* < 0.0001]. 62.3% of patients were re-hospitalized within 6 months.

**Conclusion:**

In this retrospective multicenter study, we showed that patients with NSTI required complex management and are major consumers of care. Two-thirds of them underwent a complicated hospital course, associated with a higher SAPS II score, a polymicrobial NSTI and a bacteremia.

**Supplementary Information:**

The online version contains supplementary material available at 10.1186/s13613-022-01087-5.

## Background

Necrotizing skin and soft tissue infections (NSTIs) are rare, life-threatening bacterial infections resulting in extensive tissue necrosis. NSTIs can affect any part of the body but the extremities—particularly the lower limbs—are most frequently involved [1–5]. Patient risk factors to develop NSTIs include comorbidities, such as diabetes mellitus, obesity, cardiovascular disease, intravenous drug use, and immunosuppression [1–3]. Infection can spread after traumatic injuries, including minor breaches of the skin or mucosa and even non-penetrating soft tissue injuries [1]. Hospital mortality ranges from 9.3 to 29.3%, with disabling sequelae for 30% of survivors [1, 6, 7]. Morbidity among survivors includes potential amputations and profound impact on long-term health-related quality of life [3, 8, 9]. Initial misdiagnosis is frequent [10], leading to a delayed surgical debridement of infected tissues, one of the main modifiable prognostic factors [11].

Management of patients during the acute phase of disease requires a multimodal approach of NSTIs, coordinating multiple urgent interventions, including initiation of broad-spectrum antibiotic therapy, rapid surgical debridement of all infected tissues and, when present, treatment of associated organ failures in the intensive care unit (ICU). The time to first surgical debridement is one of the main modifiable prognostic factors, which was associated with a significantly lower mortality rate when surgery was performed within six hours of hospital admission in a recent meta-analysis [12]. High-volume centers, caring for at least three patients per year, may also contribute to improving prognosis [13].

Although the acute phase management of NSTIs has been described in several observational studies, the care pathway of patients with NSTI is poorly known. Better describing the care trajectory of these patients and being able to identify patients at risk of a complicated course, requiring prolonged hospitalization, multiple surgical re-interventions, or ICU readmission, is an essential prerequisite to improve their care. The main objective of this retrospective multicenter cohort study including critically ill patients diagnosed with NSTI was to describe the hospital care pathway of these patients and identify those at risk of complicated hospital trajectory.

## Patients and methods

### Study design and patients

We conducted a multicenter retrospective cohort study including all consecutive adult patients (≥ 18 years) admitted to 13 Assistance Publique—Hôpitaux de Paris hospitals for surgically confirmed NSTI from January 1st 2015 to December 31st 2019. Patients admitted in ICUs during the inclusion period were identified using the French national hospital database (Program for Medicalization of Information Systems) with the International Classification of Diseases (ICD) diagnostic codes for “necrotizing skin and soft tissue infections”. Patients were excluded if no proof of NSTI was found during the surgical exploration. Macroscopic appearance of tissues during surgery (i.e., swollen, dull gray with a thin, brownish exudate with or without necrosis) was used in the main inclusion criterion as it is the gold standard for diagnosis of NSTI in the most recent guidelines [14]. Patients were also excluded if NSTI was not the main admission diagnosis upon medical chart review, if the localization was not known, in case of cervico-facial NSTI, or in case of an ischemic gangrene. Cervico-facial NSTIs (i.e., NSTIs caused by odontogenic infections, ear–nose–throat portals of entry, including tonsillar abscess, parotiditis, otitis media, or head and neck surgery, etc.) were not included because they have different clinical presentation and require different management strategies than NSTIs of other locations.

The primary objective of the current study was the description of the hospital care trajectory for the whole NSTI population and for NSTI subgroups defined a priori: immunocompromised vs non-immunocompromised patients and NSTI patients with abdomino-perineal vs. limb topography. Immunocompromised patients were defined as patients with solid or hematological cancer not in remission (< 2 years), neutropenia (blood neutrophils < 1500/mm^3^), HIV patients and patients taking long-term immunosuppressive drugs, including but not limited to corticosteroids. Abdomino-perineal and limb NSTIs were defined based on their anatomic topography. We analyzed the mortality rates, the total length of stay in the ICU and in hospital, the time between admission and the first surgical debridement, the rates of organ failures, the number of surgical debridement performed, the frequency of skin graft recovery, the time between the first debridement and skin graft recovery, and the rates of ICU readmission and in the hospital in the next 6 months.

Secondary objectives were to evaluate factors associated with ICU mortality and factors associated with a “complicated hospital course”, as defined by one of the following: a duration of hospital stay ≥ 6 weeks, a number of debridement performed ≥ 3, or hospital death.

Patients received information during hospital stay that data abstracted from their medical charts could be used for research purposes. Data were anonymized and compiled according to the requirements of the Commission Nationale Informatique et Liberté and the study was approved by the Institutional Review Board of *the Conseil Scientifique et Ethique de l’Entrepôt de Données de Santé de l’AP-HP* (CSE-19-34) on March 13th 2020. The study has been reported according to the STROBE guidelines regarding observational cohort studies.

### Data collection

The selection of patients was based on data from the *Programme de Médicalisation des Systèmes d'Information* (PMSI) on the diagnoses of NSTI, NSTI as principal diagnosis (PD), related diagnosis (RD) or significant associated diagnosis (SAD) using ICD-10 codes.

The first patient stay with a diagnosis of NSTI (principal, related or significant associated), admitted to the ICU with a date of admission between 2015 and 2019 was selected for the study. Identification of stays was obtained from the Health Data Warehouse of the AP-HP. This Data Warehouse aggregates all data from inpatients and outpatients of AP-HP hospitals, including socio-demographics, clinical summaries, biological data, medication prescriptions, and medical charts with diagnoses and interventions related to the hospital stays, and vital status at hospital discharge.

The ICD-10 codes used to identify NSTI were as follows: M7260, M7261, M7262, M7263, M7264, M7265, M7266, and M7267. Only hospital stays for which the patient had surgery were considered. A second check was made by rereading the reports to exclude atypical cases. Finally, a systematic manual review of each report was carried out with the collection of the following data using a Redcap electronic case report form: demographic data; main comorbidities; data relating to patients’ initial management and NSTI characteristics; patient follow-up and management during ICU stay; and ICU mortality and in-hospital mortality, re-hospitalization within 6 months after discharge, and re-hospitalization in ICU within 6 months after discharge.

### Statistical analysis

The characteristics of the study population are presented as mean (± standard deviation), or median (quartile 1; quartile 3) for continuous variables, or as number (%) for categorical variables. The comparison of characteristics between subgroups of patients was performed, depending on the conditions of application, by Student or Mann–Whitney tests for quantitative variables and by Chi-square tests or Fisher's exact test for qualitative variables.

The annual NSTI case volume per center was quantified and centers were categorized according to the threshold previously proposed by Audureau et al. [13] (i.e., centers managing < 3 or ≥ 3 NSTI cases/year). To study the outcome called “complicated hospital course”, we first used a mixed logistic regression model, entering a random effect at the center level to account for the correlation among patients treated at the same hospital. As the results showed no center effect (*p* = 0.47), the random effect was ignored. Thus, factors associated with a "complicated trajectory of care" were identified using uni- and multivariable logistic regression analyses. Variables with a *p* value < 0.20 in univariable analysis were included in multivariable analyses. The final multivariable model was obtained after gradual removal of non-significant factors in the model. Statistical analyses were performed were performed with Python software and R software (version 2.4.3, The R Project for Statistical Computing, Vienna, Austria). A *p* value < 0.05 was considered statistically significant.

## Results

### General characteristics of NSTI patients

**Fig. 1:**
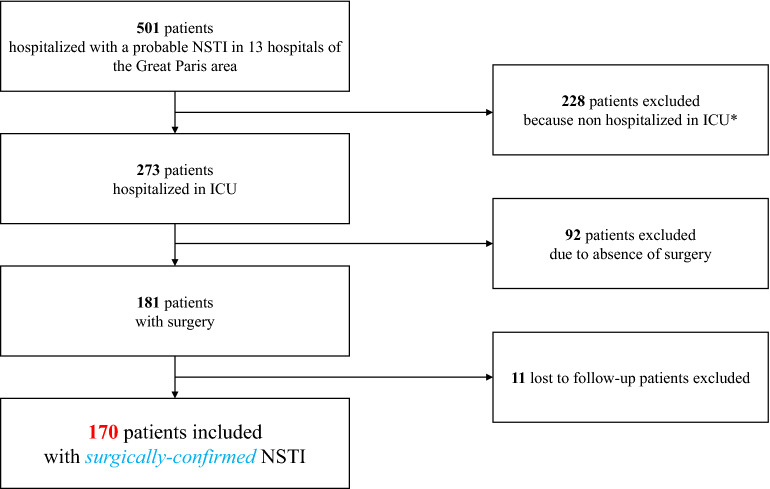
Study flowchart. *NSTI* necrotizing soft tissue infection; *ICU* intensive care unit; *These patients were men in 61.5% of cases, had a median age of 64 years (49–77) and an 11.8% in-hospital mortality (data available for 187/228 patients)

Among the 501 patients hospitalized for NSTI in 13 hospitals between January 1, 2015 and December 31, 2019, 273 required ICU admission. After manual check, 181 patients underwent surgical intervention and were thus eligible for study inclusion. Eleven of these patients were lost to follow-up, leaving 170 patients available for study inclusion (Fig. 1).

There was a predominance of male patients (71.8%), with a median age of 59 years. These patients frequently had comorbidities, including diabetes (55.3%), obesity (35.3%), chronic alcoholism (28.8%), immunosuppression (25.3%), chronic heart failure (22.4%), peripheral vascular disease (15.3%), COPD (10.1%), cirrhosis (8.9%), and end-stage renal failure (2.9%) (Table 1). 22% of the patients had taken NSAIDs before hospitalization. NSTIs were localized to the lower limbs in 72% of cases, upper limbs in 14% of cases, and to the abdomino-perineal region in 19% of cases (8 patients had multifocal NSTI).

**Table 1:** Characteristics of patients with necrotizing soft tissue infections (*n* = 170) at intensive care unit admission

	*N*	Total
		*N* = 170
Demographic data		
Age	170	59 [53–68.8]
Sex	170	
Male		122 (71.8)
Female		48 (28.2)
NSTI characteristics		
Hospital-acquired NSTI	170	38 (22.4)
Topography		
Upper limb	170	24 (14.1)
Lower limb	170	121 (71.2)
Abdominal/perineal	170	33 (19.4)
Multifocal	170	8 (4.7)
Comorbidities		
Immunosuppression^a^	170	43 (25.3)
Cancer	43	14 (32.5)
Neutropenia	43	5 (11.6)
HIV infection	43	4 (9.3)
Long-term corticosteroids	43	26 (60.5)
PAOD	170	26 (15.3)
Diabetes	170	94 (55.3)
Chronic heart failure	170	38 (22.4)
COPD	169	17 (10.1)
Cirrhosis	169	15 (8.9)
Chronic hemodialysis	170	5 (2.9)
Chronic alcohol intoxication	170	49 (28.8)
NSAIDs use before hospitalization	168	37 (22.0)
Obesity	170	60 (35.3)
Microbiological data		
Number of isolated bacteria	156	2 [1–3]
NSTI categorization	154	
Type II (monomicrobial)		65 (42.2)
Type I (polymicrobial)		89 (57.8)
Positive blood culture	170	50 (29.4)
Isolation of multi / highly resistant bacteria	168	29 (17.3)
Severity scores and organ failures		
SAPS II	161	46 [30–63]
SOFA	161	8 [4–12]
Organ failures within the first 24 h of ICU admission	163	115 (70.6)
Invasive mechanical ventilation support	163	55 (33.7)
Vasopressor support	163	113 (69.3)
Renal replacement therapy	163	35 (21.5)
Post-surgical ICU admission	163	48 (29.5)

Qualitative variables are shown as number (percentages) and continuous variables as median [quartile 1-quartile 3]

*PAOD* peripheral artery occlusive disease, *HIV* human immunodeficiency virus, *COPD* chronic obstructive pulmonary disease, *NSAIDs* non-steroidal anti-inflammatory drugs, *NSTI* necrotizing soft tissue infection, *SAPS* Simplified Acute Physiology Score, *SOFA* Sequential Organ Failure Assessment score

^a^The sum of the subgroups can be greater than 100% because patients can accumulate several criteria

Consistent with the previously reported microbiology of NSTIs [3, 4], 57.5% of NSTIs were polybacterial and 22.4% were hospital-acquired. Surgical site samples were positive in 90.6% of cases and blood cultures in 29.4% of cases. The main bacteria isolated included Enterobacteriaceae (35.7%), *Streptococcus pyogenes* (24.7%), other streptococcus species (30.5%), non-fermenting gram-negative bacteria (17.5%) and anaerobic bacteria (9.7%) (Additional file 1: Table S1). Immunosuppressed patients had more frequent non-fermenting gram-negative bacteria (35.0% vs. 11.4%, *p* = 0.002) and less streptococcus (10.0% vs. 37.7%, *p* = 0.002) isolated than others, while patients with abdomino-perineal NSTIs more frequently had *Escherichia coli* (38.5 vs. 12.5, *p* = 0.003), *Proteus mirabilis* (19.2% vs. 4.7%, *p* = 0.021) and streptococcus isolated than others.

Within the first 24 h of ICU admission, 70.6% of patients presented at least one organ failure, including 69.3% of patients who required vasopressor support, 33.7% invasive mechanical ventilation support, and 21.5% renal replacement therapy. 29.4% of patients were admitted in ICU for post-surgery care (Table 1).

### Care trajectory data

More than half (52.4%) of the patients required inter-hospital transfers before the first surgical debridement. The median time elapsed between hospital admission and first debridement was 1 day. The median time interval between hospital admission and ICU admission was 1 (0–2) day. The median duration of ICU and hospital stay were 8 and 37 days, respectively. In predefined subgroups, patients with abdomino-perineal NSTIs had a non-significantly lower duration of stay in the ICU [5 (2–19) vs. 9 (4–17) days for other locations; *p* = 0.207], and a non-significantly longer duration of stay in the hospital [39 (16–71) vs. 36 (14–69) days; *p* = 0.762]. The duration of ICU stay was not significantly different according to immunocompromised status either [9 (2–15) days in immunocompromised patients vs. 8 (4–17) days in others; *p* = 0.500] and there was a non-significant trend towards a longer total duration of hospitalization in non-immunocompromised patients [39 (16–71) vs. 23 (9–63) days; *p* = 0.070] (Table 2).

**Table 2:** Care trajectory in patients with necrotizing soft tissue infections (*n* = 170) according to immunosuppression and abdomino-perineal location status

	N	Total	Other locations	Abdomino-perineal NSTIs	*P*-value	No immunosuppression	Immunosuppression	*P*-value
		*N* = 170	*N* = 137	*N* = 33		*N* = 127	*N* = 43	
Hospital trajectory data								
Length of ICU stay (days)	170	8 [3–17]	9 [4–17]	5 [2–19]	0.207	8 [4–17]	9 [2–15]	0.500
Length of hospital stay (days)	170	37 [14–71]	36 [14–69]	39 [16–71]	0.762	39 [16–71]	23 [9–63]	0.070
Delay admission surgery (days)	168	1 [0 – 2]	1 [0 – 2]	1 [0 – 1]	0.495	1 [0–2]	1 [1–5]	**0.004**
Annual NSTI procedural volume	170				0.601			0.638
< 3/year		38 (22.3)	29 (21.2)	9 (27.3)		30 (23.6)	8 (18.6)	
≥ 3/year		132 (77.6)	108 (78.8)	24 (72.7)		97 (76.4)	35 (81.4)	
Inter-hospital transfer	168				0.934			0.484
No		80 (47.6%)	65 (48.1)	15 (45.5)		62 (49.6)	18 (41.9)	
Yes		88 (52.4%)	70 (51.9)	18 (54.5)		63 (50.4)	25 (58.1)	
Type of structure after ICU	168				**0.045**			**0.034**
Medical ward		85 (50.6)	74 (54.8)	11 (33.3)		58 (46.0)	27 (64.3)	
Rehabilitation		10 (6.0)	9 (6.7)	1 (3.0)		10 (7.9)	0	
Plastic surgery		16 (9.5)	13 (9.6)	3 (9.1)		15 (11.9)	1 (2.4)	
Other surgical ward		57 (33.9)	39 (28.9)	18 (54.5)		43 (34.1)	14 (33.3)	
Management during hospital course								
Mechanical ventilation	166	86 (51.8)	69 (51.5)	17 (53.1)	0.999	63 (50.8)	23 (54.8)	0.791
Vasopressor support	167	129 (77.2)	105 (77.8)	24 (75.0)	0.918	97 (77.6)	32 (76.2)	0.999
Renal replacement therapy	166	46 (27.7)	38 (28.4)	8 (25.0)	0.872	30 (24.2)	16 (38.1)	0.123
Organ failure	167	134 (80.2)	110 (81.5)	24 (75.0)	0.561	101 (80.0)	34 (81.0)	0.999
Skin grafting	167	54 (32.3)	46 (34.1)	8 (25.0)	0.437	46 (36.8)	8 (19.0)	0.053
Skin grafting-first surgery (days)	36	28 [13 – 39]	28 [13 – 39]	29 [18 – 43]	0.999	25 [13 – 38]	34 [31 – 40]	0.348
Amputation	166	26 (15.7)	24 (17.9)	2 (6.3)	0.174	22 (17.7)	4 (9.5)	0.307
Negative pressure therapy	167	62 (37.1)	46 (34.3)	16 (48.5)	0.191	51 (40.8)	11 (26.2)	0.131
Number of additional debridements	166	1 [0–3]	1 [0–3]	3 [1–6]	** < 0.001**	2 [0–3]	1 [0–2]	**0.041**
Outcomes								
ICU Mortality	170	37 (21.8)	30 (21.9)	7 (21.2)	0.999	21 (16.5)	16 (37.2)	**0.009**
Complicated hospital course^a^	169	123 (72.8)	97 (71.3)	26 (78.8)	0.518	89 (70.6)	34 (79.1)	**0.382**
In-hospital mortality	170	49 (28.8)	40 (29.2)	9 (27.3)	0.996	26 (20.5)	23 (53.5)	** < 0.001**
Number of debridements > 3	166	62 (37.3)	18 (13.4)	13 (40.6)	** < 0.001**	27 (21.8)	4 (9.5)	0.126
Hospital stay > 6 weeks	170	156 (91.8)	62 (45.3)	16 (48.5)	0.889	61 (48.0)	17 (39.5)	0.430
Re-hospitalization within 6 months of ICU discharge	106	66 (62.3)	49 (57.6)	17 (81.0)	0.085	56 (62.9)	10 (58.8)	0.963
ICU readmission within 6 months	89	9 (10.1)	7 (9.3)	2 (14.3)	0.628	7 (9.6)	2 (12.5)	0.662

Qualitative variables are shown as number (percentages) and continuous variables as median [quartile 1-quartile 3]

*ICU* intensive care unit, *NSTI* necrotizing soft tissue infection

^a^defined by one of the following: a duration of hospital stay ≥ 6 weeks, a number of debridement performed ≥ 3, or hospital death

Bolded variables are significant at the *p* < 0.05 level

During the course of ICU stay 80.2% of patients developed organ failures with 51.8% of patients requiring invasive mechanical ventilation, 77.2% needing vasopressor support and 27.7% renal replacement therapy. According to the predefined subgroups, there was no difference regarding initial clinical severity or organ failures during ICU stay.

Regarding surgical management, 69.9% of patients required more than one surgical debridement. Abdomino-perineal NSTIs required more surgeries than others [3 (1–6) vs. 1 (0–3) re-interventions, *p* < 0.001] (Table 2). Non-immunocompromised patients were also significantly more often re-operated than immunocompromised ones for further debridements [2 (0–3) vs. 1 (0–2) re-interventions, *p* = 0.041].

After the acute phase, 32.3% of patients required a remote skin graft, after a median time of 28 days (13–39) after the first surgical debridement, with no significant difference within both predefined subgroups. Consistent with previous series [8], 15.7% of patients underwent amputation.

At ICU discharge, more than half of the patients were transferred to a medical department (50.6%), 43.4% to a surgical department, including 9.5% to plastic surgery, and 6.0% to rehabilitation. Immunocompromised patients were significantly more often transferred to medical wards after ICU discharge (81.5% vs. 46.7%, *p* = 0.011) than non-immunocompromised patients (Table 2).

The ICU and hospital mortality rates were 21.8% and 28.8%, respectively. Strikingly, 62.3% of patients (*n* = 66/106) were re-hospitalized within 6 months of ICU discharge, including 10.1% of patients (n = 9/89) needing ICU readmission. 42.4% (*n* = 28/66) of re-hospitalizations were NSTI-related. There was no significant difference between patients with abdomino-perineal NSTIs and others in terms of in-hospital or ICU mortality, or regarding the frequency of readmissions within 6 months. Yet, immunocompromised patients showed significantly higher ICU and in-hospital mortality rates than non-immunocompromised patients (37.2% vs. 16.5%, *p* = 0.009, and 53.5% vs. 20.5%, *p* < 0.001).

### Factors associated with a complicated hospital course

A complicated hospital course, defined by in-hospital death, or prolonged length of stay (> 6 weeks), or the need for multiple re-intervention/debridement (> 3), occurred in 27.2% of patients (*n* = 46/169). The variables associated with a complicated hospital course were identified by univariable and multivariable logistic regression (Table 3). In multivariable analysis, polybacterial infection, bacteremia, and a high SAPS II score were associated with a complicated hospital course.

**Table 3:** Univariable and multivariable logistic regression analyses of factors associated with a complicated hospital course^a^

	*N*	Univariable analysis				Multivariable analysis	
		Non complicated course	Complicated course	OR [95% CI]	*P*-value	aOR [95% CI]	*P*-value
		*N* = 46	*N* = 123				
Demographic data							
Age	169	59 [52–69]	60 [53–68]	1.00 [0.98; 1.03]	0.724		
Sex	169						
Male		31 (67.4)	89 (72.4)	1.32 [0.63; 2.75]	0.459		
Female		15 (32.6)	34 (27.6)	Ref			
Hospital trajectory data							
Annual NSTI procedural volume < 3/year	169	5 (10.9)	33 (26.8)	3.01 [1.09; 8.26]	**0.033**		
Type of structure after ICU	167				0.139		
Medical ward		19 (41.3)	66 (54.6)	Ref.			
Rehabilitation		5 (10.9)	5 (4.1)	0.29 [0.08; 1.10]	0.069		
Plastic surgery		7 (15.2)	9 (7.4)	0.37 [0.12; 1.13]	0.080		
Other surgical ward		15 (32.6)	41 (33.9)	0.79 [0.36; 1.72]	0.548		
Inter-hospital transfer	167						
No		18 (39.1)	61 (50.4)	Ref			
Yes		28 (60.9)	60 (49.6)	0.63 [0.32; 1.26]	0.194		
NSTI characteristics							
Hospital-acquired NSTI	169	12 (26.1)	26 (21.1)	0.76 [0.35; 1.67]	0.494		
Topography							
Upper limb	169	7 (15.2)	17 (13.8)	0.89 [0.34; 2.32]	0.817		
Lower limb	169	32 (69.6)	88 (71.5)	1.10 [0.52; 2.31]	0.801		
Abdominal/perineal	169	7 (15.2)	26 (21.1)	1.49 [0.60; 3.72]	0.390		
Multifocal	169	0	2 (1.6)	–			
Comorbidities							
Immunosuppression	169	9 (19.6)	34 (27.6)	1.57 [0.69; 3.60]	0.286		
PAOD	169	6 (13.0)	20 (16.3)	1.29 [0.48; 3.46]	0.607		
Cancer	169	5 (10.9)	9 (7.3)	0.65 [0.20; 2.04]	0.459		
Neutropenia	169	2 (4.4)	3 (2.4)	0.55 [0.09; 3.40]	0.520		
HIV infection	169	0	4 (3.3)	–			
Diabetes	169	23 (50.0)	71 (57.7)	1.37 [0.69; 2.69]	0.369		
Chronic heart failure	169	11 (23.9)	26 (21.1)	0.85 [0.38; 1.91]	0.698		
COPD	168	5 (10.9)	12 (9.8)	0.89 [0.30; 2.70]	0.843		
Cirrhosis	168	2 (4.4)	13 (10.7)	2.62 [0.57; 12.11]	0.216		
Chronic hemodialysis	169	0	5 (4.1)	–			
Chronic alcohol use	169	8 (17.4)	41 (33.3)	2.38 [1.01; 5.55]	**0.046**		
Previous NSAIDs use	167	14 (30.4)	23 (19.0)	0.54 [0.25; 1.16]	0.115		
Obesity	169	13 (28.3)	47 (38.2)	1.57 [0.75; 3.28]	0.231		
Microbiological data							
Number of isolated bacteria	156	1 [1, 2]	2 [1–4]	1.38 [1.07; 1.78]	**0.014**		
NSTI categorization	154						
Type II (monomicrobial)		24 (55.8)	41 (36.9)	Ref.		Ref.	
Type I (polymicrobial)		19 (44.2)	70 (63.1)	2.16 [1.06; 4.41]	**0.035**	3.18 [1.37; 735]	**0.007**
Positive blood culture	169	6 (13.0)	44 (35.8)	3.71 [1.46; 9.45]	**0.006**	3.29 [1.14; 9.52]	**0.028**
Isolation of multi / highly resistant bacteria	167	3 (6.5)	26 (21.5)	3.92 [1.13; 13.67]	**0.032**		
Hospital-acquired NSTI	169	12 (26.1)	26 (21.1)	0.76 [0.35; 1.67]	0.494		
Severity scores							
SAPS II	161	33 [22–45]	53 [33.5–68]	1.05 [1.02; 1.07]	** < 0.001**	1.05 [1.02; 1.07]	** < 0.001**
SOFA	161	4 [1–7]	9.5 [5–12]	1.09 [1.01; 1.17]	**0.022**		

Qualitative variables are shown as number (percentages) and continuous variables as median [quartile 1-quartile 3]

*PAOD* peripheral artery occlusive disease, *HIV* human immunodeficiency virus, *COPD* chronic obstructive pulmonary disease, *NSAIDs* non-steroidal anti-inflammatory drugs, *NSTI* necrotizing soft tissue infection, *SAPS* Simplified Acute Physiology Score, *SOFA* Sequential Organ Failure Assessment score, *OR* odds ratio, *aOR* adjusted odds ratio

^a^Defined by one of the following: a duration of hospital stay ≥ 6 weeks, a number of debridement performed ≥ 3, or hospital death; Area under the ROC curve = 0.796; model calibration (*p* = 0.442, Hosmer–Lemeshow test)

Bolded variables are significant at the *p* < 0.05 level

## Discussion

In this retrospective multicenter study, using a large data warehouse, we focused on adult patients hospitalized in intensive care units for surgically confirmed NSTIs. Our study aimed to evaluate the care trajectory of critically ill NSTI patients and identify the factors associated with a complicated hospital course. We report key figures depicting the hospital care trajectory of patients managed in the ICU for skin and soft tissue infections: (1) An inter-hospital transfer was necessary for 52.4% of patients; (2) The median time from admission to first surgical debridement was 1 day; (3) The median duration of ICU and total hospital stay were 8 and 37 days, respectively; (4) 69.9% of the patients were re-operated with a median number of 1 additional debridement performed; (5) 32.3% of the patients required a remote skin graft, performed after a median time lag of 28 days after the first surgical debridement; (6) The mortality rates in the ICU and in the hospital were 21.8% and 28.8%, respectively; and (7) 62.3% of the patients were re-hospitalized within 6 months, including 10.1% in the ICU.

Our study confirms the heterogeneity found in previous studies regarding the comorbidities, and the clinical and microbiological features of patients with NSTIs. Our patients, consistent with previous studies [15, 16], carried numerous comorbidities, in particular diabetes (55.3%) and peripheral vascular disease (15.3%). 25.3% of the patients in our population were also immunosuppressed. This is higher than reported in previous studies, which reported a frequency of immunosuppression of 13% [17] or 16% [9], but closer to more recent studies (22.9%) [18]. In the latter study, a significantly higher mortality was found in immunocompromised patients than in others (39.1% versus 19.4%). Such findings are consistent with ours, as immunocompromised patients showed significantly higher ICU and in-hospital mortality rates than non-immunocompromised patients (37.2% vs. 16.5%, *p* = 0.009, and 53.5% vs. 20.5%, *p* < 0.001). Immunocompromised patients had significantly more infection with non-fermenting Gram-negative bacteria, but less frequent *Streptococcus* species and *Enterococcus faecalis* and may have an atypical clinical and biological presentation, often without fever or hyperleukocytosis [18].

Before and after hospital discharge, NSTI patients are important consumers of hospital care. Indeed, 52.4% of our patients required hospital transfers before surgery, with a median time from admission to the first surgical debridement of 1 day. It was unfortunately not possible to calculate this time period in hours, which is a limitation of our study. It is controversial whether inter-hospital transfer, which may be associated with a prolonged delay before the first surgical debridement, is a risk factor for mortality or, in the contrary, may be beneficial to patients. In previous studies inter-hospital transfers were not harmful, providing the volume of patients managed per center was sufficiently high. In such cases, in contrast, referring patients to these high-volume centers was associated with a benefit on mortality [13, 19]. In the current study, patients managed in high volume centers (i.e., caring for ≥ 3 NSTI patients/year) were less likely to have a complicated hospital course in univariable analysis, but there was no more significant relationship after multivariable adjustment. A previous retrospective study underlined the interest of a multidisciplinary management in a center having an expertise in this rare, severe, and complex diagnostic condition [7]. Grouping patients in reference centers that manage a high volume of NSTI cases per year, with specialized and experienced personnel, could optimize management, essentially the time to diagnosis and surgery [8, 13]. However, the optimal annual procedural case volume remains to be defined and a recent study suggested a threshold of ≥ 8 patients/year/center, which was higher than the one we considered in the current study possibly explaining the discrepant results [19].

The importance of time to surgery has been demonstrated in numerous retrospective studies [20] and is one of the most important modifiable risk factor. More recently, Kobayasshi et al. showed an increase in morbidity and number of surgical debridements associated with delayed management [21]. A meta-analysis of more than 1000 patients found a significant decrease in mortality for delays of less than 6 h and 12 h before surgery [12].

NSTI patients frequently require repeated surgeries. In our selected population, all patients had surgical debridement, per inclusion criteria. Interestingly, about 70% of the patients were returned to the operating room for additional surgery with a median number of additional debridement performed of 1 for the whole cohort, increasing to 3 in the subgroup of patients with abdomino-perineal NSTIs. Indeed, it has already been reported that Fournier’s gangrene requires more surgeries, as reported by Chawla et al. [22] with 3.5 debridement, and confirmed by Czymek et al. [23], with up to 4 debridements required. These large and disfiguring wounds form mutilating scars, and sometimes require amputations (15.7% of our population, consistent to previous literature 13.7% [9] and 18.4% [8], in two recent studies). About a third (32.3%) of the patients required a remote skin graft, within an average of 28 days after the first surgical debridement. At ICU discharge, 43.4% of survivors were transferred to surgical wards, and 50.6% to medical wards. Surprisingly, although NSTIs have been shown to lead to functional deficits and to greatly affect the quality of life of survivors, only 6% of patients were transferred to rehabilitation centers [8].

The median duration of stay in hospital and in the ICU were 37 and 8 days, respectively, consistent with previous literature [9], 24. Importantly, these durations of hospital stay are longer than those reported for patients with non-NSTI-related septic shock, having a median stay ranging from 9 to 18 days in the hospital, and 7 to 9 days in the ICU [25–27], pointing out the heavy burdens of NSTIs in terms of hospitalization. Moreover, 62.3% of patients were re-hospitalized within 6 months, including 10.1% in intensive care. The re-hospitalizations are higher than the data in the literature for non-NSTI-related septic shock [28].

In our study, the mortality rates in the intensive care unit and in the hospital were, respectively, 21.8% and 28.8%, consistent with previous literature [9, 29]. We wished to define a patient-centered and clinically meaningful endpoint in the context of NSTI management. We therefore used a composite criterion grouping an in-hospital death or a total length of stay > 6 weeks or a number of re-interventions > 3. Although we acknowledge that these thresholds may be debatable, these were chosen because they were considered clinically relevant to select a subgroup of patients with a particularly severe hospital course. We identified several risk factors for such a complicated course, including a polybacterial infection, a bacteremia with positive blood cultures, and a high SAPS 2 severity score on ICU admission.

This work has several limitations, the first of which being its retrospective design, limiting the access to available data and the generalization of its results, making our results exploratory. The selection of patients based on International Classification of Diseases 10 codes, which is sometimes very specific, may also be at the origin of a selection bias. The composite primary endpoint combines the occurrence of several developments and thus may constitute an interpretation bias, as these developments do not have the same incidence or severity.

The strengths of our work are as follows. We were able, with the support of the AP-HP data warehouse, to establish a large multicenter cohort of patients hospitalized for NSTIs in the ICU. The number of patients included, the multicentric design of the cohort, and the relatively short inclusion period (5 years) during which medical practices have changed little, have made it possible to obtain original epidemiological data enriching our knowledge of the care trajectory of these patients. The determinants of a complicated course have been identified. This study has good internal validity, as all patients had a definite and definitive diagnosis of NSTI, confirmed by surgical exploration and intraoperative findings; thus, at low risk of selection bias.

In conclusion, patients with NSTI required complex management and are major consumers of care. Two-thirds of them will undergo a complicated hospital course, associated with a higher SAPS II score, a polymicrobial NSTI and a bacteremia.

## Supplementary Information

**Additional file 1: Table S1.** Microbiological isolates obtained from 154 patients with necrotizing soft tissue infections for whom per-operative skin culture and blood culture results were available compared between predefined subgroups.

## Data Availability

The datasets used and/or analyzed during the current study are available from the corresponding author on reasonable request.

## Abbreviations

NSTIs: Necrotizing skin and soft tissue infections
ICU: Intensive care unit
ICD: International classification of diseases
AP-HP: Assistance publique des hôpitaux de Paris
PMSI: Programme de Médicalisation des Systèmes d’Information
PD: Principal diagnosis
DR: Related diagnosis
SAD: Significant associated diagnosis
PAOD: Peripheral artery occlusive disease
HIV: Human immunodeficiency virus
COPD: Chronic obstructive pulmonary disease
NSAIDs: Non-steroidal anti-inflammatory drugs
OR: Odds ratio
aOR: Adjusted odds ratio
