# Supplementary material for: Healthcare trajectory of critically ill patients with necrotizing soft tissue infections: a multicenter retrospective cohort study using the clinical data warehouse of Greater Paris University Hospitals
Source: Ann Intensive Care. 2022 Dec 20;12:115. doi: 10.1186/s13613-022-01087-5 (PMC9768077; doi:10.1186/s13613-022-01087-5)
Supplement: Supplementary file 1 — Additional file 1: Table S1. Microbiological isolates obtained from 154 patients with necrotizing soft tissue infections for whom per-operative skin culture and blood culture results were available compared between predefined subgroups. [file 13613_2022_1087_MOESM1_ESM.docx]

**Table S1.** Microbiological isolates obtained from 154 patients with necrotizing soft tissue infections for whom per-operative skin culture and blood culture results were available compared between predefined subgroups.

| **Isolated bacteria** | **All patients** | **Other locations** | **Abdomino-perineal NSTIs** | **P-value** | **No immunosuppression** | **Immunosuppression** | **P-value** |
| --- | --- | --- | --- | --- | --- | --- | --- |
|  | **N=154** | **N=128** | **N=26** |  | **N=114** | **N=40** |  |
| **Gram-negative bacteria** |  |  |  |  |  |  |  |
| Enterobacteriaceae | 55 (35.7) | 43 (33.6) | 12 (46.2) | 0.320 | 40 (35.1) | 15 (37.5) | 0.935 |
| *Eschericha coli* | 26 (16.9) | 16 (12.5) | 10 (38.5) | **0.003** | 17 (14.9) | 9 (22.5) | 0.392 |
| *Enterobacter cloacae* | 6 (3.9) | 6 (4.7) | 0 | 0.590 | 5 (4.4) | 1 (2.5) | >0.999 |
| *Klebsiella pneumoniae* | 10 (6.5) | 8 (6.3) | 2 (7.7) | 0.677 | 7 (6.1) | 3 (7.5) | 0.720 |
| *Proteus mirabilis* | 11 (7.1) | 6 (4.7) | 5 (19.2) | **0.021** | 10 (8.8) | 1 (2.5) | 0.290 |
| *Citrobacter spp* | 5 (3.3) | 4 (3.1) | 1 (3.9) | >0.999 | 4 (3.5) | 1 (2.5) | >0.999 |
| *Klebsiella oxytoca* | 3 (1.9) | 3 (2.3) | 0 | >0.999 | 3 (2.6) | 0 | 0.568 |
| *Morganella morganii* | 3 (1.9) | 3 (2.3) | 0 | >0.999 | 3 (2.6) | 0 | 0.568 |
| *Serratia marcescens* | 4 (2.6) | 4 (3.1) | 0 | >0.999 | 2 (1.8) | 2 (5.0) | 0.277 |
| Non-fermenting bacteria | 27 (17.5) | 24 (18.8) | 3 (11.5) | 0.572 | 13 (11.4) | 14 (35.0) | **0.002** |
| *Pseudomonas aeruginosa* | 25 (16.2) | 22 (17.2) | 3 (11.5) |  | 11 (9.7) | 14 (35.0) |  |
| *Acinetobacter spp.* | 4 (2.6) | 4 (3.1) | 0 |  | 4 (3.5) | 0 |  |
| **Gram-positive bacteria** |  |  |  |  |  |  |  |
| *Streptococcus pyogenes* | 38 (24.7) | 35 (27.3) | 3 (11.5) | 0.146 | 31 (27.2) | 7 (17.5) | 0.312 |
| Other streptococcus species | 47 (30.5) | 36 (28.1) | 11 (42.3) | 0.231 | 43 (37.7) | 4 (10.0) | **0.002** |
| *Streptococcus agalactiae* | 10 (6.5) | 8 (6.3) | 2 (7.7) | 0.677 | 8 (7.0) | 2 (5.0) | >0.999 |
| *Streptococcus anginosus* | 12 (7.8) | 8 (6.3) | 4 (15.4) | 0.121 | 12 (10.5) | 0 | **0.037** |
| *Streptococcus constellatus* | 5 (3.3) | 1 (0.8) | 4 (15.4) | **0.003** | 4 (3.5) | 1 (2.5) | >0.999 |
| *Streptococcus oralis* | 4 (2.6) | 1 (0.8) | 3 (11.5) | **0.015** | 4 (3.5) | 0 | 0.573 |
| *Streptococcus dysgalactia* | 5 (3.3) | 5 (3.1) | 0 | 0.590 | 5 (4.4) | 0 | 0.328 |
| *Enterococcus faecium* | 8 (5.2) | 7 (5.5) | 1 (3.9) | >0.999 | 6 (5.3) | 2 (5.0) | >0.999 |
| *Enterococcus faecalis* | 14 (9.1) | 10 (7.8) | 4 (15.4) | 0.258 | 14 (12.3) | 0 | **0.021** |
| *Staphylococcus aureus* | 39 (25.3) | 36 (28.1) | 3 (11.5) | 0.127 | 33 (29.0) | 6 (15.0) | 0.125 |
| *Staphylococcus epidermidis* | 7 (4.6) | 6 (4.7) | 1 (3.9) | >0.999 | 5 (4.4) | 2 (5.0) | >0.999 |
| *Corynebacterium spp* | 11 (7.1) | 9 (7.0) | 2 (7.7) | >0.999 | 8 (7.0) | 3 (7.5) | >0.999 |
| **Anaerobic bacteria** | 15 (9.7) | 9 (7.0) | 6 (23.1) | 0.022 | 11 (9.7) | 4 (10.0) | >0.999 |

Results are shown as n (percentages); p values come from the chi square or the Fisher test, as appropriate;

**Bolded** values are significant at the p<0.05 level.
